# PG I and PG II show unique value in diagnosing postoperative biochemical recurrence in patients with gastric cancer after total gastrectomy

**DOI:** 10.1007/s12672-024-01091-0

**Published:** 2024-06-17

**Authors:** Jiuru Zhang, Jiameng Liu, Liyang Dong, Xi Wang, Xueqian Mao, Yufei Mao, Chaoming Mao

**Affiliations:** 1 Department of Nuclear Medicine, The Affiliated Hospital of Jiangsu University, Jingkou District, No. 438 Jie Fang Road, Zhenjiang, 212000 Jiangsu People’s Republic of China; 2 Department of Ultrasound Medicine, The Affiliated Hospital of Jiangsu University, Jingkou District, No. 438 Jie Fang Road, Zhenjiang, 212000 Jiangsu People’s Republic of China

**Keywords:** Gastric cancer, Postoperative biochemical recurrence, Total gastrectomy, Pepsinogen (PG), Group I pepsinogen (PG I), Group II pepsinogen (PG II)

## Abstract

**Objective:**

To investigate the potential of group I pepsinogen (PG I) and group II pepsinogen (PG II) as diagnostic markers for recurrence in gastric cancer (GC) patients post-total gastrectomy.

**Methods:**

Ninety-six patients who underwent total gastrectomy for GC between June 2022 and June 2023 were included in this study. Clinical data, serum samples, and ascites samples were collected. Patients were categorized based on recurrence status at the time of sample collection and the primary tumor site. PG I and PG II levels were determined using a chemiluminescent immunoassay, and their clinical utility following total gastrectomy for GC was evaluated via receiver operating characteristic (ROC) curve analysis.

**Results:**

This study included 96 GC patients who underwent total gastrectomy, 55 of whom experienced postoperative recurrence (57.29%). The levels of serum PG I (27.86 (27.04, 30.97) vs. 26.05 (24.16, 27.09) ng/mL; *P* < 0.0001) and PG II (1.95 (1.23, 3.05) vs. 0.63 (0.47, 0.90) ng/mL; *P* < 0.0001) were significantly greater in the recurrent group compared to the non-recurrent group. The secretion of PG I and/or PG II by metastatic cancer cells correlated with the primary lesion site. When the cut-off value for serum PG I was 26.93 ng/mL, the area under the curve (AUC) for PG I was 0.77. When the cut-off value for serum PG II was 0.96 ng/mL, the AUC reached 0.90. The combined AUC was 0.97.

**Conclusion:**

These findings suggest that serum PG I and PG II are valuable biomarkers for identifying GC patients with biochemical recurrence post-total gastrectomy.

## Introduction

Pepsinogen (PG) is an inactive enzyme precursor that is synthesized and released by major cells within the gastric mucosa, and it is categorized into two groups: group I pepsinogen (PG I) and group II pepsinogen (PG II) [1, 2]. Chief cells and mucous neck cells in the gastric fundus and body glands predominantly secrete PG I, making it one of the most abundant pepsinogens. The pyloric glands, cardiac glands, and Brunner's glands produce PG II, albeit in lesser quantities, and it plays a secondary role in the digestive process compared to PG I [3, 4]. Most PG is delivered into the stomach cavity, and only a fraction (approximately 1%) traverses the capillaries of the gastric mucosa and enters the circulation. Circulating PG exhibits remarkable stability, and fluctuations in its levels serve as an evaluative measure of the quantity of gastric mucosal glandular cells and their secretory function [5, 6].

Notably, measurements of serum PG I and PG II levels serve as diagnostic tools for evaluating stomach health and assessing cancer risk. The levels of PG I and PG II, and the PG I/PG II ratio, offer valuable insights into gastric mucosal damage, chronic gastritis, and the risk of cancer development [7, 8]. When the stomach is entirely resected, PG I and PG II concentrations decrease significantly within 1 week [1]. Therefore, the production of detectable PG I and/or PG II may be sensitive and specific indicators of biochemical recurrence in patients who have undergone total gastrectomy.

CA19-9 may be useful for detecting the recurrence of gastric cancer after total gastrectomy [9, 10]. Other tumor markers, such as CEA, CA72-4, CA50, and CA242, are also used for the diagnosis and staging of gastric cancer [11–13]. However, their specificity and sensitivity are generally low [14]. Notably, our study demonstrated that PG I and PG II levels effectively diagnosed biochemical recurrence after radical total gastrectomy for gastric cancer, which provides a sensitive, rapid, and cost-effective method of dynamic follow-up. This measurement serves as a theoretical basis for the monitoring of biochemical recurrence in patients with gastric cancer after total resection, which is similar to the clinical value of serum thyroglobulin in papillary thyroid carcinoma after total thyroidectomy [15].

## Materials and methods

### Patients and study design

In this comprehensive retrospective investigation, we meticulously scrutinized the medical records of patients who underwent total gastrectomy for gastric cancer at the Affiliated Hospital of Jiangsu University in Zhenjiang, China. The study period was spanned from June 2022 to June 2023. Eligible participants were aged between 46 and 80 years. The following inclusion criteria were used: (1) histological confirmation of gastric cancer; (2) radical total gastrectomy at our institution; and (3) complete postoperative hematological and biochemical data. The following exclusion criteria were used: (1) received chemotherapy or immunotherapy; (2) had severe cardiac, hepatic, renal or other organ insufficiency combined with other immune-related diseases; (3) had intestinal polyps or infectious gastrointestinal diseases; or (4) had other malignant tumors. This retrospective study obtained full approval from the Ethics Committee at the Affiliated Hospital of Jiangsu University (Approval number: KY2023K0603) and adhered to the ethical principles outlined in the Declaration of Helsinki with utmost diligence. All patients provided informed consent.

### Serum sample collection

Blood samples from all postoperative follow-up subjects were taken in the morning on an empty stomach. Whole blood samples were obtained using standard venipuncture techniques and collected in serum separator tubes. The serum was separated from the tube contents via centrifugation at 4000× gravity for 10 min. Serum samples were detected within 12 h in the clinical laboratory.

### Extraction and cultivation of malignant ascites-derived tumor cells

Thoracic or abdominal fluid (100 mL) from metastatic gastric cancer patients was extracted under sterile conditions. Heparin sodium anticoagulant (10 U/mL for thoracic/abdominal fluid) was used, followed by centrifugation at 1200 rpm/min for 5 min. The supernatant was discarded, and the pellet was resuspended in Hank's solution. A combination technique using 100% Ficoll and 75% Ficoll was applied to separate cancer cells from the thoracic/abdominal fluid (centrifuged at 2000 rpm/min for 20 min). The isolated gastric cancer cells were cultured in 10% 1640 medium for 24 h, and the culture supernatant was collected for PG level analysis.

### Pepsinogen detection

Serum PG I and PG II levels were detected using a commercial chemiluminescent immunosandwich assay according to the manufacturer’s instructions (Snibe Co. Ltd., Shenzhen, China). Briefly, ABEI and FITC were labeled with monoclonal antibodies against PG I or PG II, respectively, and mixed with the samples to form immune complexes. The supernatant was removed, and the precipitated complexes were washed 3 times with wash solution. The instrument automatically pumps in substrate solutions 1 and 2 and automatically monitors the relative light unit (RLU) emitted within 3 s. The concentration of PG I or PG II is proportional to the RLU, and the detection instrument automatically calculates the concentration of PG I or PG II. Data analysis involved five-parametric curve fitting, and assay controls included kit standards and multiplex controls. Technical personnel from SNIBE oversaw these experiments.

### Statistical analysis

Statistical analyses and graph creation were performed using GraphPad Prism 8.0 (GraphPad Software Inc.). Data normality was assessed using the Kolmogorov–Smirnov and Shapiro–Wilk tests. Normally distributed data are expressed as means ± SD, and t tests were used for comparisons between groups. Non-normally distributed data are presented by medians (upper digit to lower digit), and comparisons between groups were tested using the Mann–Whitney U test. Comparisons of count data were performed using the chi-squared test. The receiver operating characteristic (ROC) curve method was used to determine the sensitivity and specificity of various diagnostic approaches. A *P* value less than 0.05 was considered statistically significant.

## Results

### Basic patient data

**Fig. 1:**
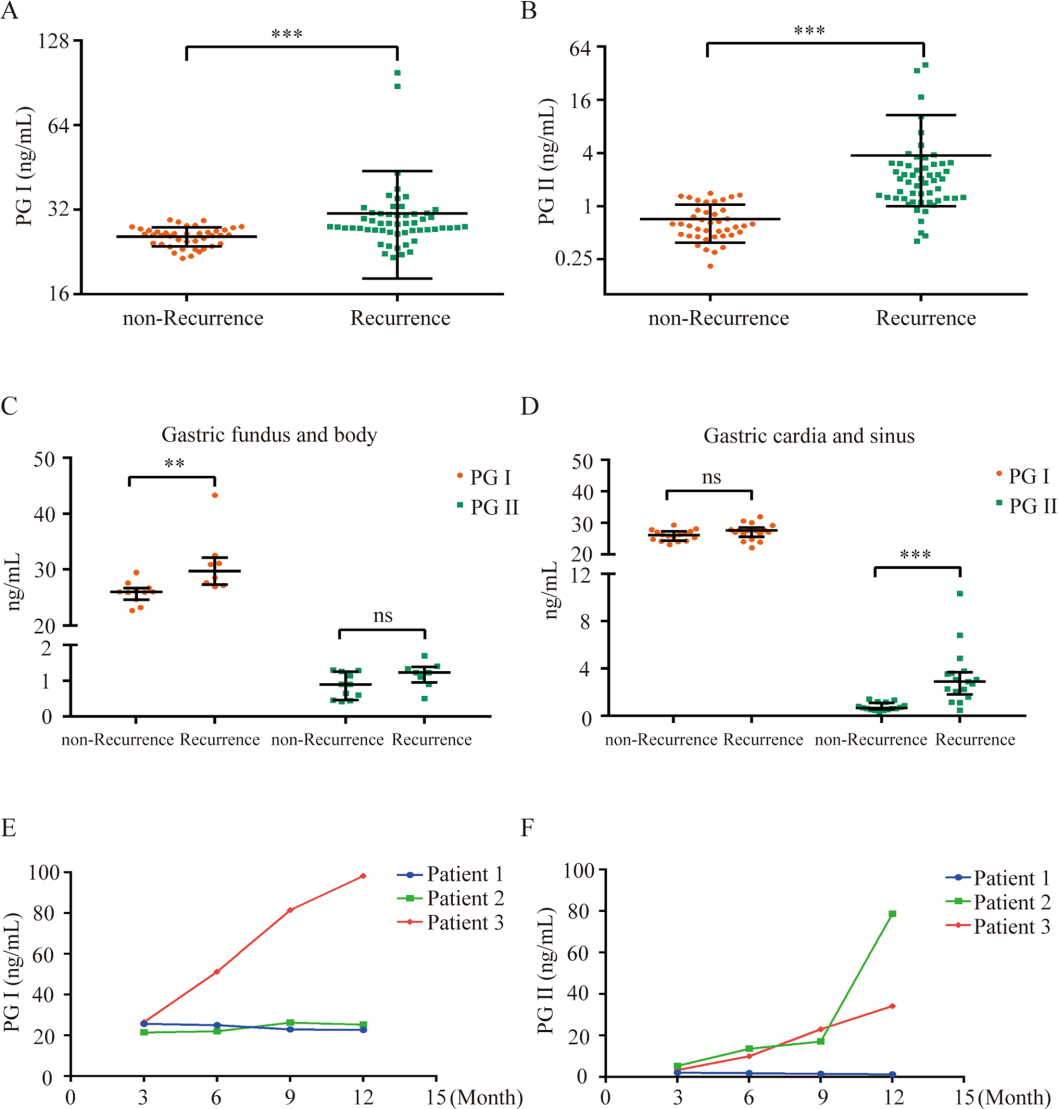
Elevated Serum PG I and PG II Levels in Postoperative Recurrence of Gastric Cancer Patients with Total Gastrectomy. The serum levels of PG I and PG II were detected using chemiluminescence immunoassays in gastric cancer patients who underwent total gastrectomy. **A** Differences in serum PG I levels between patients who experienced postoperative recurrence (n = 55) and those who did not (n = 41) are shown. **B** The differences in serum PG II levels between patients with (n = 55) and without (n = 41) postoperative recurrence are shown. **C** The variations in serum PG I and PG II levels are shown between patients with primary tumor occurrence in the gastric body and fundus (n = 8) and patients without recurrence (n = 11). **D** The differences in serum PG I and PG II levels between patients with primary tumors in the gastric sinus and cardia (n = 17) and patients without recurrence (n = 16) are shown. The dynamic changes are depicted in serum PG I levels (**E**) and PG II levels (**F**) at 3, 6, 9 and 12 months following surgery in three patients, one who did not experience recurrence (blue), and two who did have recurrence (red and green). Comparisons between groups were tested using the Mann–Whitney U test. Significance levels are denoted as *P < 0.05, **P < 0.01, and ***P < 0.001

From June 2022 to June 2023, a total of 96 gastric cancer (GC) patients who met the study's inclusion criteria were enrolled at the Affiliated Hospital of Jiangsu University. The mean age of the participants was 64.97 ± 7.59 years, and male patients comprised 73.96% (71/96) of the cohort. Postoperative recurrence was observed in 55 of the 96 GC patients who underwent curative surgery, which represented 57.29% of the total population. Notably, the serum PG levels were notably higher in the postoperative recurrence group than the non-recurrence group (Table 1; Fig. 1A, B).

**Table 1:** Patient characteristics at baseline

Characteristics	Total	Non-Recurrence	Recurrence	*P*
Number	96	41	55	
Age (year)	64.97 ± 7.59	63.71 ± 7.80	65.91 ± 7.36	0.1607
Sex				0.8170
Male, n (%)	71 (73.96)	31 (75.61)	40 (72.73)	
Female, n (%)	25 (26.04)	10 (24.39)	15 (27.27)	
PG I (ng/mL)	27.15 (25.11, 29.19)	26.05 (24.16, 27.09)	27.86 (27.04, 30.97)	< 0.0001
PG II (ng/mL)	1.2 (0.63, 2.20)	0.63 (0.47, 0.90)	1.95 (1.23, 3.05)	< 0.0001

Data were expressed as means ± SD, n (%) or medians (upper digit to lower digit). PG I, group I pepsinogen; PG II, group II pepsinogen

### Secretion of PG I and PG II by gastric cancer cells

To provide evidence that serum PG is secreted by gastric cancer cells, ascites from three advanced gastric cancer (AGC) patients were collected. Tumor cells were meticulously extracted from the ascites for subsequent cell culture. PG I and PG II concentrations in the serum, ascites and culture supernatant of cancer cells were measured to confirm the capacity of tumor cell secretion of gastric proteases by detecting the content of gastric protease precursors in the culture supernatant. As shown in Table 2, PG I and PG II were detectable at high levels in the culture supernatant of primary gastric cancer cells extracted from the ascites of AGC patients, which is consistent with the ascites results. These findings suggest that gastric cancer cells directly secreted PG I and PG II.

**Table 2:** Secretion of PG in metastatic gastric cancer cells

Patients	Serum		Ascites		Culture supernatant	
	PG I (ng/mL)	PG II (ng/mL)	PG I (ng/mL)	PG II (ng/mL)	PG I (ng/mL)	PG II (ng/mL)
1	–	–	32.09	> 100	25.2	> 100
2	40.68	21.47	36.26	> 100	30.05	11.96
3	> 520	> 100	> 520	64.92	33.98	29.5

PG I: group I pepsinogen; PG II: group II pepsinogen

### Elevated serum PG I and PG II levels are associated with postoperative recurrence in gastric cancer patients who undergo total gastrectomy

To examine whether PG levels were a viable biological marker for recurrence following total gastric cancer resection, we performed comprehensive statistical analyses of PG I and PG II levels in GC patients who had undergone total gastrectomy and compared these levels in patients with and without gastric cancer recurrence. Our analysis revealed significant differences in serum PG between the recurrent and non-recurrent groups. We observed a substantial increase in serum PG I level in the recurrent group compared to the non-recurrent group (Table 1; Fig. 1A, 27.86 (27.04, 30.97) vs. 26.05 (24.16, 27.09) ng/mL; *P* < 0.0001). Similarly, PG II levels were markedly elevated in the recurrent group compared to the non-recurrent group (Table 1; Fig. 1B, 1.95 (1.23, 3.05) vs. 0.63 (0.47, 0.90) ng/mL; *P* < 0.0001).

To determine the relationship between pepsinogen levels and the site of tumorigenesis, patients in both groups were categorized based on the primary tumor location, the gastric body and fundus group and the sinus and cardia group. In the gastric body and fundus group, there was a significant increase in PG I level in the recurrent group compared to the non-recurrent group (29.75 (27.33, 32.16) vs. 26.05 (24.65, 26.72) ng/mL; *P* = 0.0008). The PG II level was also elevated in the recurrent group, but this difference did not reach statistical significance. Conversely, there was a significant increase in PG II level in the recurrent group compared to the non-recurrent group in the gastric sinus and cardia group (2.89 (1.82, 3.71) vs. 0.65 (0.54, 1.09) ng/mL; *P* < 0.0001). The PG I level was elevated in the recurrent group, but this difference was not statistically significant (Fig. 1C, D; Table 3). Collectively, these findings support the potential of PG as an indicator of postoperative biochemical recurrence in gastric cancer patients.

**Table 3:** Differences in PG I and PG II secretion in patients with postoperative recurrence from different primary sites of gastric cancer

Group	PG (ng/mL)	Total	Non-Recurrence	Recurrence	*P*
Gastric fundus and body	PG I	26.97 (25.96, 29.47)	26.05 (24.65, 26.72)	29.75 (27.33, 32.16)	0.0008
	PG II	1.10 (0.60, 1.28)	0.90 (0.46, 1.26)	1.23 (0.95, 1.38)	0.0860
Gastric cardia and sinus	PG I	26.88 (24.77, 27.84)	26.13 (24.43, 27.31)	27.59 (25.65, 28.56)	0.1388
	PG II	1.19 (0.63, 2.97)	0.65 (0.54, 1.09)	2.89 (1.82, 3.71)	< 0.0001

Data were expressed as medians (upper digit to lower digit). PG I: group I pepsinogen; PG II: group II pepsinogen

We closely monitored the postoperative PG I and PG II levels of three patients 3, 6, 9 and 12 months after surgery (two with recurrence and one without). As depicted in Fig. 1E, F, we observed a gradual upward trend in PG I and PG II levels in patients who experienced recurrence. One of these patients exhibited a significant simultaneous increase in PG I and PG II. In contrast, patients without recurrence exhibited relatively stable levels of PG I and PG II, with no discernible changes. The observed changes in PG levels in these three patients further supported the role of PG as a valuable biological diagnostic indicator for recurrence and non-recurrence. PG provides a marker for postoperative follow-up of patients with gastric cancer after total gastrectomy.

### Diagnostic potential of PG I and PG II for postoperative biochemical recurrence in gastric cancer patients who underwent total gastrectomy

**Fig. 2:**
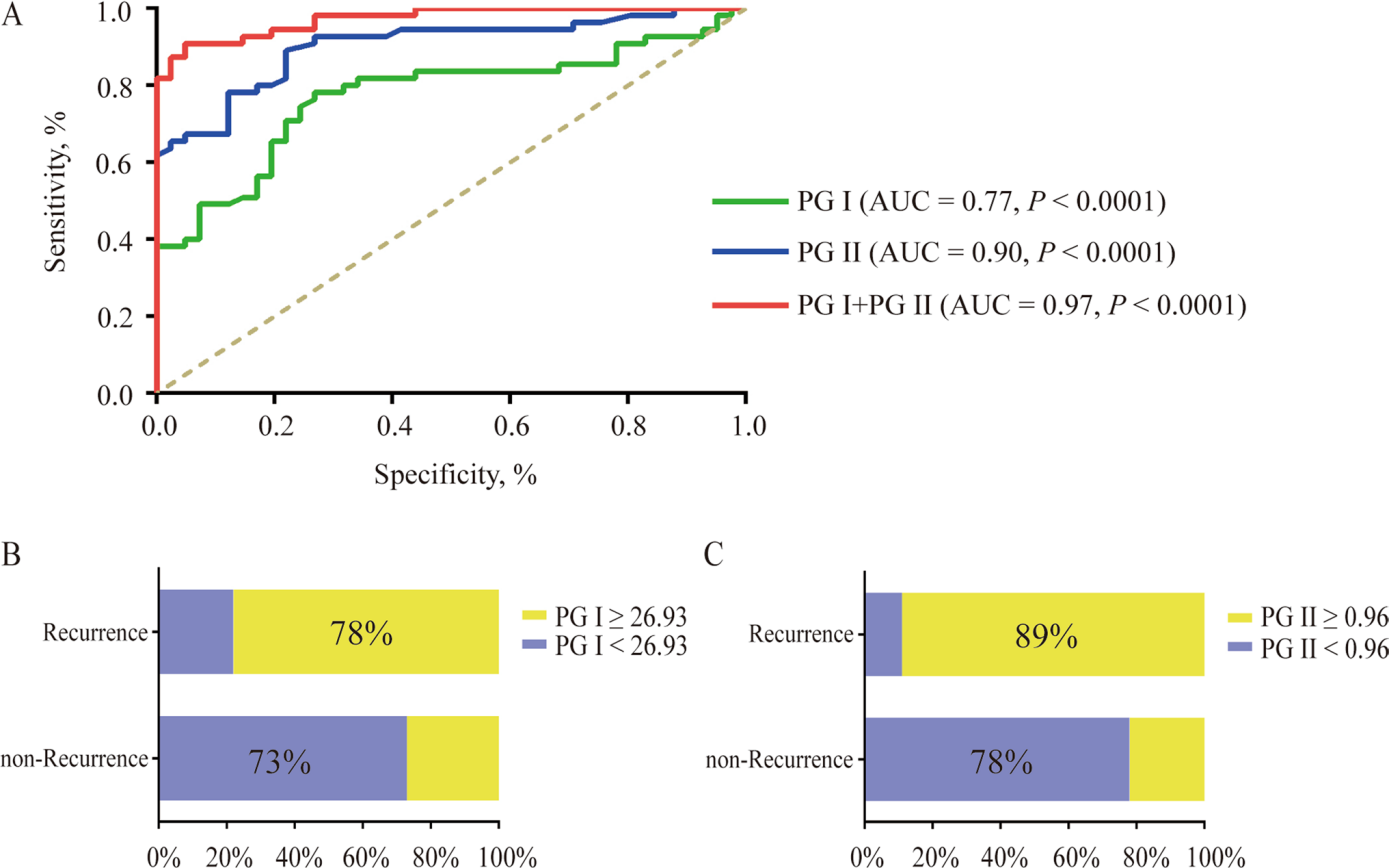
Diagnostic Potential of PG I and PG II for Postoperative Recurrence in Gastric Cancer Patients with Total Gastrectomy. **A** The receiver operating characteristic (ROC) curves for PG I, PG II and their combination were generated, and the area under the curve (AUC) was analyzed. The percentages of patients with serum PG I (**B**) and PG II (**C**) expression in the postoperative recurrence group are compared to the non-recurrent group, as defined by the optimal cut-off value. Significance levels are indicated as *P < 0.05, **P < 0.01, and ***P < 0.001

To assess the diagnostic value of PG levels in determining postoperative biochemical recurrence in GC patients who underwent total gastrectomy, we used ROC curves for PG I, PG II, and their combination (Fig. 2A and Table 4). When the cut-off value for PG I was 26.93 ng/mL, the area under the curve (AUC) for PG I was 0.77 (*P* < 0.0001), with a sensitivity of 0.78 and a specificity of 0.73. When the cut-off value for PG II was 0.96 ng/mL, the AUC for PG II was 0.90 (*P* < 0.0001), with a sensitivity of 0.89 and a specificity of 0.78. Subsequent analyses assessed whether the combination of PG I and PG II further enhanced the sensitivity and specificity of diagnosing postoperative biochemical recurrence. The ROC curves for the combination of PG I and PG II yielded an AUC of 0.97 (*P* < 0.0001) and exhibited a remarkable sensitivity of 0.91 and a specificity of 0.95. These results support the high diagnostic value of PG I and PG II for distinguishing postoperative recurrent and non-recurrent patients.

**Table 4:** ROC analysis of PGI and PGII in the diagnosis of postoperative recurrence

Variables	PG I	PG II	PG I + PG II
Cut-off (ng/mL)	26.93	0.96	–
AUC (95% CI)	0.77 (0.68–0.87)	0.90 (0.84–0.96)	0.97 (0.95–0.99)
Sensitivity	0.78	0.89	0.91
Specificity	0.73	0.78	0.95
PPV	0.80	0.84	0.72
NPV	0.71	0.84	1.00
Youden Index	0.51	0.67	0.86
*P*	< 0.0001	< 0.0001	< 0.0001

ROC: receiver operating characteristic; AUC: area under the curve; PPV: positive predictive value; NPV: negative predictive value; PG I: group I pepsinogen; PG II: group II pepsinogen

We further examined the proportion of patients with high and low PG levels in the recurrence and non-recurrence groups using the defined cut-off values. As depicted in Fig. 2B, C, using the optimal cut-off values, 73% of patients in the non-recurrent group had PG I levels below 26.93 ng/mL, and 78% had PG II levels below 0.96 ng/mL. In contrast, 78% of patients in the recurrent group had PG I levels greater than 26.93 ng/mL, and 89% had PG II levels greater than 0.96 ng/mL. These findings further emphasize that PG I and PG II may be used to effectively diagnose biochemical recurrence during the postoperative follow-up of patients with gastric cancer after total gastrectomy.

## Discussion

Gastric cancer originates from the epithelial cells of the gastric mucosa, and it poses a significant health challenge due to its aggressive nature, propensity for invading adjacent organs, such as the esophagus, pancreas, and liver, and high incidence of lymph node and distant metastasis [16, 17]. Moreover, gastric cancer is the second-most common malignant tumor in China, and it constitutes approximately half of the global number of gastric cancer cases, with an upward trend [18]. Despite advancements in surgical techniques, total gastrectomy remains the primary treatment for advanced gastric cancer in clinical practice [19–21]. However, the postoperative recurrence rate remains unacceptably high, at 40% to 80%, with a dismal five-year survival rate of less than 10% [22–24]. Postoperative recurrence is a major contributor to the poor prognosis of gastric cancer patients. Therefore, vigilant monitoring of postoperative recurrence, early detection, and timely intervention are of paramount importance in extending patient survival [25, 26]. Notably, the influence of chemotherapy, targeted therapy, and immunotherapy drugs on imaging may lead to delayed diagnosis or inaccurate assessments, which affects the clinical evaluation of tumor recurrence. Therefore, it is imperative to identify a convenient, cost-effective, sensitive, and precise method for monitoring postoperative recurrence following total gastrectomy in gastric cancer patients. Although tumor markers have greater sensitivity in diagnosing tumor recurrence than other approaches, such as medical imaging, the quest for a biochemical marker with high sensitivity and specificity for the diagnosis of postoperative recurrence of gastric cancer remains elusive.

Previous studies detected PG I expression in gastric cancer tissues using immunohistochemistry [27, 28]. To determine whether gastric cancer cells directly secreted PG I or PG II, we investigated PG I and PG II levels in ascites collected from recurrent gastric cancer patients and in the culture supernatant of gastric cancer cells derived from these ascites. The results unequivocally indicated substantial levels of PG I and PG II and provided direct evidence that these metastatic gastric cancer cells secreted significant quantities of PG I and PG II. This result serves as a foundational cornerstone for the use of PG I and PG II as robust diagnostic indicators for postoperative biochemical recurrence of GC following total gastrectomy, which is analogous to the clinical value of serum thyroglobulin in predicting biochemical recurrence of papillary thyroid carcinoma following total thyroidectomy [15].

Therefore, we analyzed serum PG I and PG II concentrations in follow-up GC patients after total gastrectomy and examined their relationship with biochemical recurrence. The serum concentrations of PG I and PG II were notably increased in patients with recurrent gastric cancer post-total gastrectomy, and PG II demonstrated a particularly substantial elevation compared to PG I. This result may be attributed to the fact that non-recurrent patients who have undergone total gastrectomy often maintain a relatively high baseline level of serum PG I (< 27 ng/mL), which suggests that tissues beyond the gastric mucosa contribute to PG I secretion. Conversely, non-recurrent patients who undergo total gastrectomy tend to exhibit very low levels of serum PG II (< 1 ng/mL).

The secretion of PG I and PG II are closely linked to the anatomical location of the stomach, but whether the elevated presence of PG I and PG II in recurrent gastric cancer patients also correlate with the site of the primary lesion was not known. Therefore, we performed a comprehensive investigation to determine whether the increased levels of PG I and PG II in recurrent gastric cancer patients were associated with the location of the primary tumor. Our findings revealed intriguing patterns. There was a notable increase in serum PG I level in patients who experienced recurrence following surgery for tumors located in the gastric fundus and body. Conversely, there was no significant alteration in PG II levels. Patients who experienced recurrence after surgery for tumors located in the cardia and sinus demonstrated a marked increase in serum PG II levels, but PG I levels remained relatively stable. This observation suggested that the type of PG secreted by metastatic cancer cells was significantly associated with the primary lesion tissue location. However, notably, a considerable number of recurrent patients exhibited simultaneous increases in PG I and PG II. This finding underscores the heterogeneity and complexity of the biological behavior of gastric cancer tissue cells. This finding also supports the importance of simultaneously assessing both markers when diagnosing the biochemical recurrence of gastric cancer.

To further substantiate the link between biochemical recurrence and serum PG levels, we performed a longitudinal follow-up study of three patients. Our observations revealed a discernible pattern. Patients who remained recurrence-free after surgery consistently maintained low serum PG levels. In stark contrast, individuals who experienced recurrence exhibited a progressive increase in serum PG I and/or PG II levels. This dynamic observation supports the hypothesis that postoperative biochemical recurrence in gastric cancer patients who have undergone total gastrectomy may exhibit increased blood levels of PG I and/or PG II.

In our pursuit of clinical efficacy assessment, we used ROC curve analysis to establish optimal cutoff values. A cutoff value of 26.93 ng/mL for PG I and 0.96 ng/mL for PG II yielded results that were characterized by high specificity and sensitivity in the evaluation of postoperative biochemical recurrence in gastric cancer patients who underwent total gastrectomy. PG-I exhibited an AUC of 0.77, and PG-II had an AUC of 0.90, which supported its distinctiveness primarily in clinical efficacy. Notably, the combination of both markers achieved an impressive AUC of 0.97. These findings suggest that PG I and PG II function as exceptional biological markers for the early diagnosis of biochemical recurrence of GC following total gastrectomy. Traditional tumor markers, such as CEA, CA19-9, and CA72-4, also demonstrate commendable sensitivity in detecting biochemical recurrence in GC patients who underwent total gastrectomy [9]. However, these indicators are not highly specific or sensitive. Therefore, PG I and PG II may compensate for this deficiency due to their tissue-oriented secretion characteristics.

Although the physiological role of PG as a digestive enzyme has been recognized for many years, previous research, dating back more than three decades [29], was constrained by the limitations of the detection methods at that time, such as radioimmunoassay (RIA). These methodological constraints primarily hindered the study of PG for the diagnosis of gastric cancer biochemical recurrence, and there were no parallel investigations into the relevance of PG II. The clinical value of PG I and PG II in diagnosing postoperative biochemical recurrence in GC patients who have undergone total gastrectomy has not received the attention it deserves in clinical practice.

In the present study, we investigated the clinical value of PG in the diagnosis of biochemical recurrence among GC patients who have undergone total gastrectomy. However, this study has certain limitations. It does not provide a comprehensive clarification on the types of gastric cancer cells secreting PG, and whether all types of gastric cancer cells secrete PG is not known. The identification of recurrent patients in the present study relied on traditional indicators, including clinical data, pathological findings, and imaging studies, as opposed to the comprehensive follow-up data. Therefore, there may be a subset of recurrent patients with subtle clinical presentations that remained unconfirmed, which could affect the sensitivity of the evaluation. This factor partially explains the suboptimal sensitivity and false positives of PG in the non-recurrence group.

Another limitation is that this study was a retrospective study performed at a single institution, and it included a relatively modest number of patients who experienced recurrence. In conclusion, this investigation contributes valuable dynamic monitoring tools and molecular evaluation indicators for the early diagnosis of postoperative biochemical recurrence in GC patients who have undergone total gastrectomy. After obtaining the basic levels of PG I and PG II in follow-up patients, the monitoring of dynamic changes in PG I and PG II may allow for effective management of postoperative biochemical recurrence.

## Data Availability

All datasets used or analyzed during the current study are available from the corresponding author on reasonable request.
